# Does Intragroup Conflict Intensity Matter? The Moderating Effects of Conflict Management on Emotional Exhaustion and Work Engagement

**DOI:** 10.3389/fpsyg.2021.614001

**Published:** 2021-05-31

**Authors:** Zinat Esbati, Christian Korunka

**Affiliations:** Department of Applied Psychology, Work, Education, Economy, Faculty of Psychology, University of Vienna, Vienna, Austria

**Keywords:** intensity, relationship conflict, task conflict, work engagement, emotional exhaustion

## Abstract

To elucidate the distinct effects of relationship conflict (RC) and task conflict (TC), we investigated the intensity (low vs. high) of the two types of conflict on emotional exhaustion and work engagement. Furthermore, we examined how cooperative vs. competitive conflict-handling styles moderate the relationship between the two types of conflict and emotional exhaustion and work engagement. We also examined the role of emotion regulation (cognitive reappraisal and distraction) as a covariate to control its effects on the study variables. Utilizing two separate 2 × 2 between-subject experimental designs, we recruited 120 employees from several companies in Austria. The results suggest that higher levels of both RC and TC are positively related to emotional exhaustion and negatively to work engagement. A cooperative conflict management style moderated the effects of both RC and TC on work engagement. The results suggest decoupling RC and TC and examining the interplay between the intensity of intragroup conflict types and conflict management styles provides insights into the connection between the levels of conflict, conflict management, emotional exhaustion, and work engagement. Additionally, it supports the usage of distraction as a viable regulation strategy for managing the effects of high levels of RC on emotional exhaustion.

## Introduction

The use of workgroups—two or more people interacting interdependently toward a common goal—in organizations has steadily increased over the past few decades (Kozlowski and Bell, 2013). However, although bringing people together in a workgroup can be beneficial, individual differences and interdependencies of workgroup members (the extent to which workgroup members cooperate and work interactively to get tasks done; Stewart and Barrick, 2017) can also increase the chances of *intragroup conflict* (e.g., Korsgaard et al., 2008; De Wit et al., 2012).

Intragroup conflict is defined as perceived incompatibilities or differences among workgroup members (Jehn, 1995). This mainly originates from disagreements over task viewpoints, such as how the tasks are being performed called *task conflict* (TC) or from interpersonal incompatibilities in personality, habits, or style called *relationship conflict* (RC).

Research implies that RC is dysfunctional (Benitez et al., 2018; Esbati and Korunka, 2020) and TC is beneficial for desirable workgroup outcomes (Jehn, 1995; Tjosvold et al., 2003). RC is associated with affective responses (Jehn, 1995). TC is beneficial as it increases workgroup members' tendency to scrutinize task opinions and allows critical evaluations of each other's work viewpoints, resulting in a better understanding of the task at hand (De Dreu and Weingart, 2003; Jehn and Bendersky, 2003). However, two meta-analyses of the intragroup conflict literature (De Dreu and Weingart, 2003; De Wit et al., 2012) find no support for an overall positive association between TC and workgroup outcomes, such as performance and job satisfaction. Perhaps because RC and TC have rarely been investigated separately, little is known about the distinct effect of TC and RC on workgroup outcomes (See Korsgaard et al., 2008). The equivocal results of conflict prompted us to separate and explore these effects.

Research suggests that intragroup conflict is largely an emotional entity (Jehn, 1995), causing stress responses (De Dreu and Weingart, 2003) with behavioral, psychological, and medical consequences (Quick et al., 1997). However, studies of the effects of RC and TC on well-being indicators at work are limited (Meier et al., 2013; Kuriakose et al., 2019) although well-being can potentially develop conditions to undermine or enhance workgroup outcomes (e.g., Huynh et al., 2014; Jungst and Blumberg, 2016). Consistent with the *job demand-resource model* (Demerouti et al., 2001; Bakker and Demerouti, 2007), we chose two vital indicators of well-being: (a) emotional exhaustion (feelings of being overextended; Maslach and Jackson, 1981) and work engagement (a positive, fulfilling, work-related state of mind; Schaufeli and Bakker, 2004). Research suggests that poorer workgroup outcomes are partly the result of at least two psychological factors: (a) reductions in work engagement (e.g., Brown and Leigh, 1996) and (b) increases in emotional exhaustion (e.g., Meier et al., 2013). Both can reduce the motivation of workgroup members to attain workgroup goals. Can such reduction be minimized or avoided in the context of RC and TC?

Research suggests that the influences of RC and TC are contingent upon how members respond to or manage them (e.g., De Dreu and Weingart, 2003; De Wit et al., 2012). *Conflict management* (strategies implemented by group members to reduce conflict; DeChurch et al., 2013) is shown to be effective in handling conflict (e.g., Dijkstra et al., 2011). However, there is limited study on the moderating effects of conflict management on the relationship between TC, RC, emotional exhaustion, and work engagement.

To better understand the influences of RC and TC, we address three circumstances. First, we decoupled RC and TC as Solansky et al. (2014) contend that decoupling RC and TC is critical for understanding the effects of conflict type on individual and workgroup outcomes. We consider at least two plausible reasons for decoupling RC and TC: the possibility of the co-occurrence of RC and TC (De Wit et al., 2012) and the strong association between them (De Dreu and Weingart, 2003). Second, we address conflict intensity: the amount and frequency of conflict that could influence workgroup outcomes (Andrews and Tjosvold, 1983). Third, we address cooperative vs. competitive conflict management styles as a moderating variable under which the influence of RC and TC might change (Dijkstra et al., 2005; Benitez et al., 2018). These two conflict management strategies are both active conflict-engaging strategies (Dijkstra et al., 2009). The conflict–outcome relationship might vary across people who employ different means of regulating their emotional reactions to conflict (e.g., Curşeu et al., 2012; Griffith et al., 2014). We, thus, controlled the influence of emotion regulation via cognitive reappraisal and distraction as a covariate.

In sum, we address three gaps in the literature. Although previous research on RC and TC largely focuses on “hard” outcomes, such as performance and innovation, we focus on “soft” outcomes, namely emotional exhaustion and work engagement. Despite prior research largely examining the effects of RC and TC simultaneously, we decouple RC and TC and manipulate their intensity (high vs. low) to elucidate equivocal results of TC. Although prior research shows the effectiveness of the cooperative style of conflict management on handling conflict, its effectiveness has not been explored in different intensity levels of RC and TC and in relation with emotional exhaustion and work engagement.

### The Present Study

The first purpose of the present study was, thus, to elucidate the distinct effects of high vs. low RC and TC on emotional exhaustion and work engagement and to shed more light on the elusive effects of TC. The second purpose was to explore the moderating roles of conflict management (controlling for the role of emotion regulation) in the association between high vs. low levels of RC and TC and emotional exhaustion and work engagement. We employed an experimental study to fulfill these purposes, hoping to replicate, cross-validate, and extend previous statistical findings.

We lay out in detail an overview of the theoretical framework of the study leading to the formulation of the study hypotheses in the following section.

## Theory and Hypothesis Development

### Conflict and Emotional Exhaustion

Emotional exhaustion is a state of feeling overextended and depleted of one's emotional resources (Maslach and Jackson, 1981). Research indicates a positive correlation between conflict at work and emotional exhaustion (Spector and Bruk-Lee, 2008; Benitez et al., 2018). From a work stress perspective, RC is a threatening work stressor (Spector and Bruk-Lee, 2008), as it generates tension with the psychological costs of increasing strain and stress (Dijkstra et al., 2011; Bruk-Lee et al., 2013). Emotionally charged interactions among workgroup members generate extra emotional demands. When such demands exceed group members' resources, it leads to emotional exhaustion (Dijkstra et al., 2009). Compared with RC, TC causes less stress and strain, yet disagreements over work-related issues may threaten group members' striving for a positive self-view and may induce stress (Meier et al., 2013). Research also indicates that, as TC intensifies, arousal and tension increase (e.g., Carnevale and Probst, 1998), which may lead to a drain on energy. This might be a reason for proposing that TC “should be of moderate intensity” (De Dreu, 2008, p. 9).

Given the above, a key question is whether the effects of RC and TC on emotional exhaustion vary across the intensity (high vs. low) of conflict. We argue that high RC and TC can lead to emotional exhaustion. As Janssen et al. (1999, p. 122) state nicely, a high level of RC produces “intolerance and antagonistic attributions concerning each other's intentions and behaviors.” This may escalate emotional loads that might exceed group members' cognitive and physiological resources. Accordingly, workgroup members feel chronic fatigue and drained of energy. Consistent with this reasoning, there is a positive correlation between RC and emotional exhaustion (Benitez et al., 2018; Esbati and Korunka, 2020). Conversely, when RC is low, group members are less likely to question the motives underlying personal disagreements. The implication is that emotional and cognitive resources are available for managing conflict.

Furthermore, we argue that, as fundamental task disagreements intensify, group members' cognitive and emotional loads increase (e.g., Carnevale and Probst, 1998). This depletes most of the group members' limited resources. Emotionally taxing experiences can lead to emotional exhaustion and feelings of depletion (Maslach and Jackson, 1981). According to threat rigidity theory (Staw et al., 1981), when workgroup members constantly and strongly challenge each other's work perspectives, they may feel threatened—the feeling of losing their respect and acceptance on work viewpoints (Dickerson et al., 2004; Jehn et al., 2008). This, in turn, invokes a set of psychological and physiological responses (Dickerson, 2008). Conversely, when TC is low, disagreements are tolerable, and emotional and cognitive resources are available for managing work view discrepancies (Shaw et al., 2011). We, thus, propose the following:

Hypothesis 1: Emotional exhaustion is increased with high levels of RC as opposed to low levels.

Hypothesis 2: Emotional exhaustion is increased with high levels of TC as opposed to low levels.

### Conflict and Work Engagement

Work engagement is defined as “a positive, fulfilling, work-related state of mind, characterized by vigor, dedication, and absorption” (Schaufeli et al., 2002, p. 74). Engaged workgroup members dedicate their “full self” to work and are highly motivated to work. They also experience positive emotions. Positive emotions positively influence the intensity of work motivation or the amount of effort contributed (e.g., Staw et al., 1981), and unpleasant affective experiences produce negative motivational states (Seo et al., 2004). As negative emotions can be present with any types of conflict (Jehn, 1997), it would be beneficial to explore whether the effects of RC and TC on work engagement would vary across the intensity (high vs. low) of conflict.

There is a dearth of research on the direct effect of conflict on work engagement. However, extant studies report that RC demotivates employees (Chen et al., 2011) and is negatively related to work engagement (Selmer et al., 2013). Jungst and Blumberg (2016) also suggest that employees are less engaged in their work when they experience or perceive TC.

In the present study, we argue that the distinct influences of RC and TC on work engagement are contingent on the conflict intensity (high vs. low). When RC is highly prevalent in the workgroup, personal disagreements become more salient and reveal underlying tension (Jehn and Mannix, 2001). Consequently, the feeling of threat, i.e., social and psychological discomfort, increases within the workgroup (Seo et al., 2004). These feelings create disincentives for engaging at work. This propensity is consistent with threat rigidity theory (Staw et al., 1981) and social self-preservation (Dickerson et al., 2004), which propose that social threat activates a stress response that produces defensiveness, closed-mindedness, and either an intensification or avoidance reaction (Carnevale and Probst, 1998; O'Neill and McLarnon, 2018), which could lead to disengaging at work. Along these lines, Buric and Macuka (2018) document in a two-wave, cross-lagged analysis that teachers who experience more anger, fatigue, and hopelessness in the first measurement point were also less engaged at the second assessment. Thus, we propose that a high level of RC leads to workgroup members feeling less devoted to their workgroup and less likely immerse themselves in the work.

Building on information processing theory (Galbraith, 1974), we argue that, with heightened TC, cognitive and emotional loads also increase, thereby drawing resources away from the processing of divergent perspectives (Carnevale and Probst, 1998). De Wit et al. (2012) state that too much TC distracts cognitive resources that may not be directly invested in the task. This narrows the attentional field and creates reluctance and withdrawal (Carnevale and Probst, 1998), leading to disengaging at work. Hence, we hypothesize the following:

Hypothesis 3: Work engagement is decreased with high levels of RC as opposed to low levels.

Hypothesis 4: Work engagement is decreased with high levels of TC as opposed to low levels.

### The Moderating Role of Conflict Management Style

The literature on conflict suggests that the influence of conflict on the workgroup dynamic is not independent from the way conflict is handled by workgroup members (Dijkstra et al., 2011). According to dual concern theory (Pruitt and Rubin, 1986), reactions to disagreement arise from two motivational underpinnings: concern for the self and concern for the other party. The combination of these two dimensions yields five strategies, of which this study focuses on two: cooperative (high concern for both self and others) and competitive (high concern for self and low concern for others). We chose these two strategies because group members communicate cooperative or competitive intention to the other party in the conflict (Tjosvold et al., 2003). Furthermore, these two conflict management strategies are both active conflict-engaging strategies (Dijkstra et al., 2009).

A cooperative conflict management style is characterized by exchanging information on priorities and preferences, problem-solving, and making trade-offs between important and unimportant issues, reducing both conflict and stress (Friedman et al., 2000). Group members who handle conflict cooperatively are more likely to engage in constructive communication, leading to improved understanding of personal disagreements and improved understanding of other members' viewpoints (Dijkstra et al., 2011).

In contrast, a competitive conflict management style is characterized by threats, bluffs, and intimidation (e.g., De Dreu and Beersma, 2005). A competitive style frequently leads to fractious debate and conflict escalation. This contributes negatively to group functioning and group effectiveness (Behfar et al., 2008).

#### The Effects on Emotional Exhaustion

Given the above, we argue that a cooperative conflict management style makes the workgroup less conflict-laden. Group members can manage the feeling of threat and improve their self-worth and lessen the frustration evoked by personal discrepancies (Friedman et al., 2000; Gross and Guerrero, 2000; Tekleab et al., 2009). This can be done through defining conflicting interests and values as a mutual issue to be solved and searching for shared interests or exploring new options that address the interests of the conflicting parties. Research shows that a cooperative strategy is the most effective conflict management for RC, particularly at a high level of conflict (Andrews and Tjosvold, 1983), and mitigates the adverse effects of RC on emotional exhaustion (Medina and Benitez, 2011).

Furthermore, through the cooperative style, group members can discuss their work view differences openly, or at least actively, ask for further clarification, and consider their own and their opponent's concerns to resolve their discrepancies (see Tekleab et al., 2009). They may hold a “we are in it together” attitude, seek a solution to ensure workgroup goals are met, and treat conflict as a mutual problem to be solved. This attitude prevents conflict escalation or a negative cycle of tension among workgroup members (Jehn et al., 2008), allows mutual respect (Dijkstra et al., 2011), maintains feeling of self-worth and self-efficacy, and reduces the tension and frustration caused by TC (Tekleab et al., 2009). The reduction could mitigate the intensity of TC and its depletion of energy allowing workgroup members to focus their resources on the task at hand.

Conversely, a competitive style consumes group members' resources and results in resource loss. As disputants utilize pressure and critical remarks and may wish to outdo one another, this brings irritation to the surface and erodes emotional recourses. Thus, we hypothesize the following:

Hypothesis 5: A cooperative rather than a competitive conflict management style moderates the relationship between RC levels and emotional exhaustion.

Hypothesis 6: A cooperative rather than a competitive conflict management style moderates the relationship between TC levels and emotional exhaustion.

#### The Effects on Work Engagement

The positive impacts of a cooperative style could also apply to work engagement. Costa et al. (2014, 2015) propose that hindering or lowering RC levels facilitates the building of teamwork engagement as workgroup members are “more able to provide constructive criticism and become less self-centered and more concerned with the team's collective goal accomplishment and with the task(s) at hand” (Costa et al., 2015, p. 13). A cooperative style encourages effective communication to verbalize disagreements in a helpful and less obstructive manner and facilitates recognition of the legitimacy of each other's personal and work-related differences in perspectives. Research indicates that open-minded discussion contributes to lessen the adverse effects of TC on workgroup outcomes (Tekleab et al., 2009; Jiang et al., 2013). De Jong et al. (2013) shows that group members with high levels of openness had constructive TC. This minimizes the emotional and cognitive resource load required for work engagement. However, holding on to self-centered work views and prioritizing personal gain over all other advantages impair constructive interactions and intensify disagreements (Friedman et al., 2000), leading workgroup members to withhold their task effort (Jehn, 1995). Thus, we hypothesize the following:

Hypothesis 7: A cooperative rather than competitive conflict management style moderates the link between RC levels and work engagement.

Hypothesis 8: A cooperative rather than competitive conflict management style moderates the link between TC levels and work engagement.

#### Emotion Regulation

Emotion regulation is defined as “the processes [strategies] by which individuals influence which emotions they have, when they have them, and how they experience and express these emotions” (Gross, 1998, p. 275). One emotion regulation strategy is called antecedent-focused, and it can occur before the emotional responses have been completely developed (Gross, 1998). Cognitive reappraisal and distraction (attentional depletion) are two types of antecedent-focused strategy. Both involve taking action prior to or during an emotion experience. These two strategies are the most possible and cognitively healthy emotion regulation strategies in the context of organizations (Gross and John, 2003; Griffith et al., 2014).

Cognitive reappraisal involves the cognitive reconstruction of a potentially emotion-eliciting event such that it reduces its emotional impact (Lazarus and Alfred, 1964). Cognitive reappraisal is a flexible means of downregulating even tense negative emotions (e.g., Gross, 2001). It improves interpersonal functioning and well-being (John and Gross, 2004; Spaapen et al., 2014) through increasing positive emotions and decreasing negative emotions experienced and expressed (Matta et al., 2014).

Distraction involves a mental turning away from the emotion-generating event. Griffith et al. (2014) show that distraction rather than cognitive reappraisal moderates the negative consequences associated with RC. In a series of experiments, Sheppes and Meiran (2007) found that distraction was immediately effective in reducing negative experiences even when initiated late. By contrast, when reappraisal was initiated late, it was less successful in reducing the negative experience.

Emotion regulation strategies have different consequences in different contexts of emotion intensity (e.g., Sheppes et al., 2011). Sheppes et al. (2011) illustrate that, in the context of low-intensity emotional situations, participants were inclined to employ reappraisal rather than distraction; in the context of high-intensity emotional situations, distraction was a preferred strategy.

We argue that, when RC (or TC) is high, distraction (a) blocks emotional input early before it accumulates, and (b) cognitive and emotional resources are not restricted for further self-control (Gross, 2002). Conversely, when RC (and TC) is low, cognitive demand is low (Sheppes et al., 2014). This implies that group members have the opportunity to take a different perspective of the situation. Reframing disagreements would attenuate the negative effect of RC (and TC) on emotional exhaustion.

In sum, we address emotion regulation as a covariate in our study. Using a cognitive reappraisal strategy provides an opportunity for group members to examine and comprehend different perspectives. This makes them open to each other's ideas, values, and interests. This, in turn, encourages using a cooperative strategy to resolve conflict. Utilizing distraction can also promote cooperation as it reduces disrespectful and unpleasant interactions and may provide group members with the opportunity for detachment from negative emotions; thereby they could recover and regain their emotional and cognitive resources (see Jiang et al., 2013).

## Materials and Methods

### Participants

We estimated the sample size of this study by calculating the power size by using G^*^Power software set at 1–β = 0.95 and α = 0.05. The effect size was kept at the range of value 0.40. The power extracted was 0.95. The results estimated a sample size of 84 participants. To boost the statistical power of the study, we increased the number of participants to 120; that is, 15 participants were assigned to each condition.

Contrary to existing experimental studies in the conflict arena that use University students as participants, we recruited employees from companies in Vienna. They voluntary participated in this study.

The composition of participants in the study was as follows: age range, 23–63 years (mean [M] = 39.50 years, standard deviation [SD] = 10.53); 55.8% of the sample was female. Eighty-five (70.9%) employees held masters degrees and higher. On average, the participants reported 14.41 years (SD = 9.34) of work experience. The work experience ranged from 2 to 38 years.

### Procedure

We developed eight scenarios for the study. We recruited 120 volunteers from several companies in Austria. They were in service (e.g., consulting company), technology (e.g., computer, hardware, and software products), and international organizations (federal agencies). Due to the difficulty of data collecting from companies, we reached out to the prospective participants through the authors' personal networks. After initial contact with them and getting their informed consent, they received an invitation letter to participate in the study.

This study was completed online. To apply randomization, we assigned, each participant randomly to one of eight conditions: (1) high RC and a cooperative or (2) competitive conflict management style; (3) low RC and a cooperative or (4) competitive conflict management style; (5) high TC and a cooperative or (6) competitive conflict management style; (7) low TC and a cooperative or (8) competitive conflict management style. The questionnaire was sent to the email addresses of the participants who consented to take part in the study. The participants were assured of anonymity. They were asked first to read the script and imagine themselves as a member of the workgroup described and then answer the study questions.

### Design

**Figure 1:**
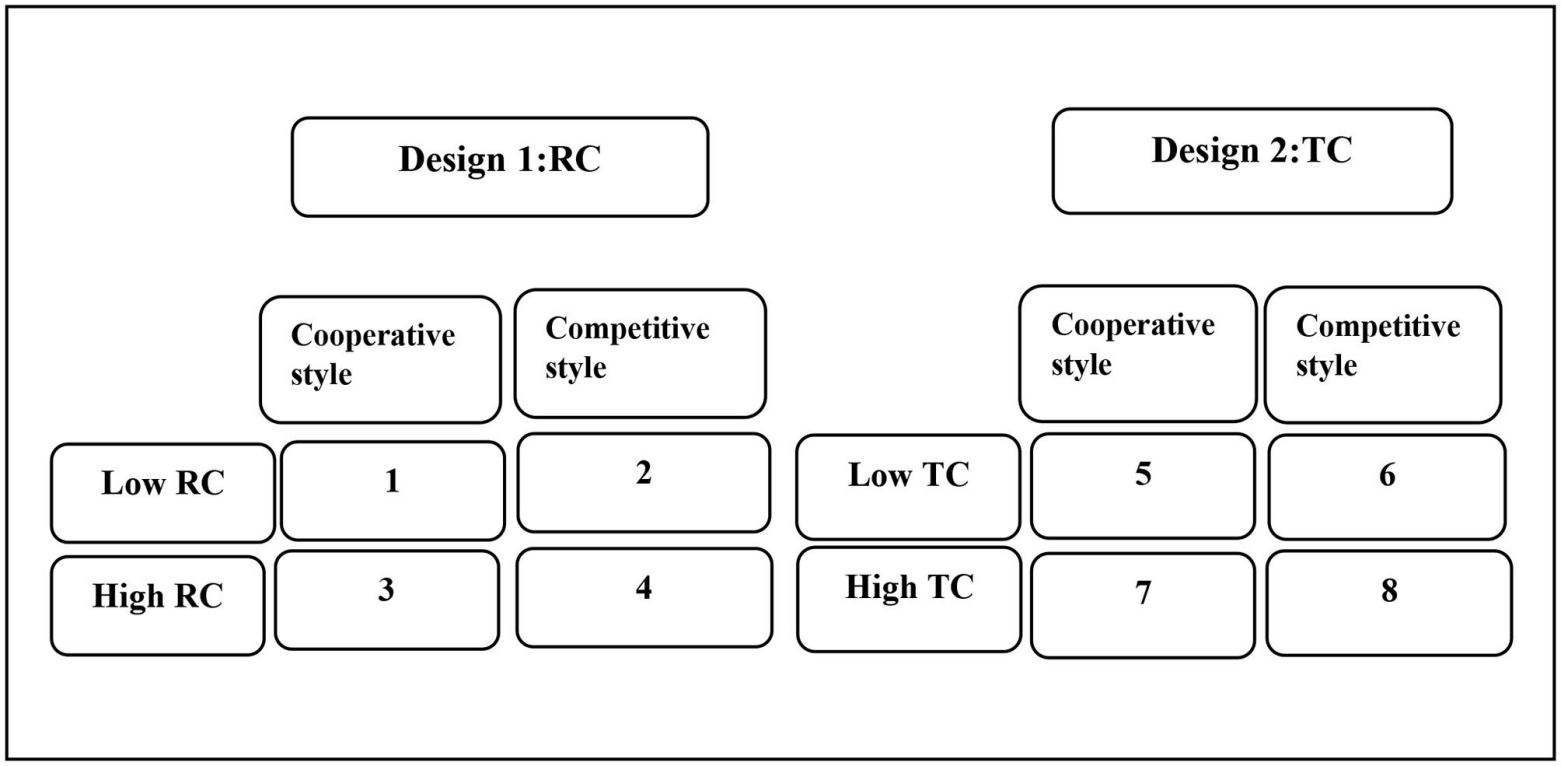
The design of the study. RC, Relationship conflict; TC, Task conflict; 1–8: each condition of the study.

Two separate 2 × 2 between-subjects designs were used to investigate the influence of conflict type (low vs. high TC, low vs. high RC) and conflict management style (cooperative vs. competitive) on work engagement and emotional exhaustion (Figure 1). We created two scenario-based experimental designs. In the first, RC intensity (low vs. high) was manipulated while the level of TC was held constant. In the second, TC intensity (low vs. high) was manipulated while the level of RC was held constant. To test the role of emotion regulation (cognitive reappraisal and distraction) for the effects on work engagement and emotional exhaustion, this variable was included as a covariate.

### Manipulation

High- or low-level TC and RC and a cooperative and competitive style of conflict management manipulations were developed for this study (Appendix A). These scripts were designed to reflect eight conditions: (a) high or low RC and a cooperative or competitive conflict management style; (b) high or low TC and a cooperative or competitive conflict management style.

First, we recruited 30 volunteers from some companies in Vienna to answer the manipulation check phase. First, participants answered questions on the covariate emotion regulation. Then, they were asked to read a scenario and imagine themselves in the situation described. The first paragraph of the scenario informed the participants about the condition of the workgroup (e.g., suppose you and four other people are all members of the same company workgroup. You are all working full time and are all at the same rank in the company). The second paragraph described the conflict situation (e.g., each of you propose very different solutions. Each member's ideas are usually challenged in heated discussions). The third paragraph addressed how the group members managed the conflict (e.g., most often, the disagreements are resolved by combining your diverse ideas into a group's consensus). Then, the participants answered the dependent variable questions.

The scenarios included several keywords to indicate the intensity of conflict and the conflict management style. The descriptions of high or low intragroup conflict and conflict management styles were consistent with the definition of intragroup conflict (Jehn, 1995) and conflict management outlined by Rahim (2002).

### Dependent Variables

#### Work Engagement

The Utrecht Work Engagement Scale (UWES; Schaufeli et al., 2002) was included for assessing work engagement. The items on the questionnaire were measured on a seven-point rating scale from 1 (strongly disagree) to 7 (strongly agree). A sample item is “Working in this workgroup is full of meaning and purpose.” Cronbach's alpha was 0.80.

#### Emotional Exhaustion

We employed the emotional exhaustion subscale of the Maslach Burnout Inventory–General Survey (MBI-GS; Schaufeli et al., 1996). The items on the questionnaire were measured on a seven-point rating scale from 1 (none) to 7 (a great deal). A sample item is “I am burned out from this workgroup.” Cronbach's alpha was 0.85.

### Covariate

Emotion regulation was assessed as a covariate. Prior research suggests that emotion regulation potentially influences work engagement and emotional exhaustion (e.g., Griffith et al., 2014; Costa et al., 2015; Esbati and Korunka, 2020). To control for this potential effect, we assessed the participants' emotion regulation, including cognitive reappraisal (Revised Emotion Regulation Questionnaire, Spaapen et al., 2014) and distraction (Esbati and Korunka, 2020). Cronbach's alpha for cognitive reappraisal and distraction were 0.81 and 0.85, respectively.

### Manipulation Checks

To check manipulation, the participants were asked four questions. Two questions assessed how the participant perceived the conflict in the workgroup. As an example, “how much task conflict do you think there is in the workgroup described above?” Two items measured how the participant perceived the conflict management. As an example, “how much of a cooperative-style conflict management do you think there is in the workgroup described above?” The questions were measured on seven-point rating scales, i.e., from 1 (none) to 7 (a great deal).

The results of the manipulation checks indicate that our manipulations worked as intended. The participants in each condition perceived expected manipulation. Participants in the TC condition rated the TC script as having TC but not RC (*M* = 4.87, SD = 1.64 vs. *M* = 0.12, SD = 0.34, *t* = [1.29] = 11.31, *p* = 0.001). Participants in the RC condition rated the RC script as having RC but not TC (M = 4.69, SD = 2.12 vs. *M* = 1.20, SD = 1.21, *t* = [1.29] = 5.58, *p* = 0.001). Participants in the cooperative conflict management style condition perceived a cooperative style in the script rather than a competitive one (*M* = 6.42, SD = 0.67, *t*_(1,29)_ = 35.59, *p* = 0.001). The same was true for perceiving a competitive style condition in the script rather than a cooperative one in the competitive conflict management style condition (*M* = 6.37, SD = 0.49, *t*_(1,29)_ = 34.60, *p* = 0.001). The responses to the manipulation check items showed that participants in both designs and all conditions differed as expected for all manipulations.

### Data Analysis

Using SPSS 24, we computed descriptive statistics, an analysis of variance (ANOVA), and a two-way ANOVA to explore the relationship of the variables of interest under high vs. low RC and TC (hypotheses 1–4). To test the proposed hypotheses, the influence of RC (and TC) levels (low vs. high) and conflict management styles (cooperative vs. competitive) on work engagement and emotional exhaustion were assessed using multivariate analysis of covariance (MANCOVA) and analysis of covariance (ANCOVA), whereas emotion regulation was retained as a covariate (hypotheses 5–8). As an additional analysis, we tested the moderating role of emotion regulation in the link between RC (and TC) on emotional exhaustion and work engagement, and we conducted moderation analysis using the SPSS PROCESS macro; Model 1, developed by Hayes (2012).

## DESIGN 1: Relationship Conflict

### Results

This was a 2 (high vs. low RC) × 2 (cooperative vs. competitive style) between-subjects design. Means, SD, and correlations were calculated to provide a general understanding of the relationship between the variables of interest (Tables 1, 2). The means and SD according to the conditions for RC, work engagement, and emotional exhaustion are presented in Table 3.

**Table 1:** Descriptive statistics, and correlations coefficients for the study variables.

**Variables**	***M***	**SD**	***N***	**1**	**2**	**3**
1. Emotional exhaustion	4.19	0.62	60	**-**		
2. Work engagement	3.94	0.67	60	−0.74^**^	**-**	
3. Emotion regulation	4.93	0.27	60	−0.27^**^	0.27^*^	**-**

** *p = 0.01*,

* *p = 0.05, two-tailed*.

**Table 2:** Descriptive statistics for relationship conflict and conflict management styles (*N* = 60).

**Variables**		**Relationship conflict**		**Conflict management style**	
		**Low**	**High**	**Cooperative**	**Competitive**
1. Emotional exhaustion	**M**	3.71	4.67	3.96	4.41
	**SD**	0.41	0.39	0.63	0.53
2. Work engagement	**M**	4.47	3.42	4.11	3.78
	**SD**	0.46	0.38	0.34	0.54

**Table 3:** Descriptive statistics according to the conditions for work engagement and emotional exhaustion (*N* = 60).

**Levels of Relationship conflict**	**Conflict management style**	**Emotional exhaustion**		**Work engagement**	
		***M***	**SD**	***M***	**SD**
High	Cooperative	4.47	0.34	3.43	0.37
	Competitive	4.85	0.34	3.40	0.40
Low	Cooperative	3.45	0.39	4.80	0.22
	Competitive	3.97	0.23	4.14	0.40

Table 2 illustrates that participants perceived emotional exhaustion significantly higher at a high RC condition vs. low [*F* = (1, 58) = 84.14, *p* < 0.01]. Compared with the low RC condition, high-RC participants reported less work engagement [*F* = (1, 58) = 94.54, *p* < 0.01]. The findings indicate that high-RC participants perceived more emotional exhaustion and less work engagement compared with their low-RC counterparts. Linear regression indicates that the level of RC is associated with emotional exhaustion (β = 0.77, *t* = 9.17, *p* = 0.001) and work engagement (β = −0.79, *t* = −9.72, *p* = 0.001). As such, a high (but not low) level of RC is associated with emotional exhaustion, and a low (but not high) level of RC is associated with work engagement. Thus, the findings support H1 and H3. Table 3 indicates that, in the conditions with a high level of RC and using a cooperative style, the mean of emotional exhaustion (*M* = 4.47, SD = 0.34) is greater than low level of RC (*M* = 3.45, SD = 0.39).

The influence of RC level (low vs. high) and cooperative vs. competitive style on work engagement and emotional exhaustion were assessed using MANCOVA and ANCOVA.

**Figure 2:**
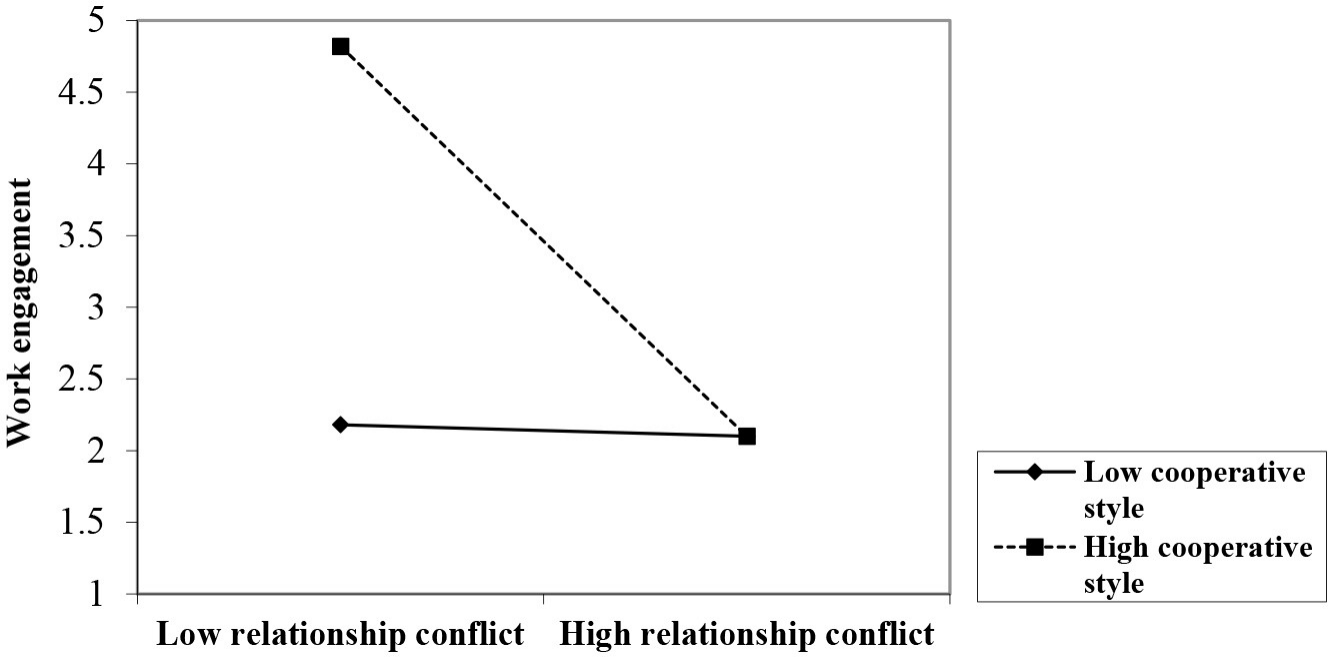
The moderating effect of conflict management style on the relationship between relationship conflict (RC) and work engagement.

To perform a MANCOVA, emotion regulation was retained as a covariate. Using Wilks' lambda, the results show a significant interaction of RC level and conflict management style on work engagement [*F* = (1, 55) = 11.72, *p* < 0.01]. The finding indicates that a cooperative conflict management style moderates the link between RC and work engagement, supporting H7 (Figure 2). The figure shows that using a high level of cooperation in a high-RC situation is not beneficial. Perhaps this is because the tension and resentment caused by such conflict restrict emotional resources. In a highly emotional situation, the restriction could create a gap between the group members' emotional arousal (e.g., high animosity) and investing their efforts in the work. The results indicate that the interaction term of RC level and conflict management style on emotional exhaustion was non-significant (ns) [*F* = (1, 55) = 0.85, *p* > 0.05]. Therefore, H5, stating the moderating role of a cooperative style on the relationship between RC level and emotional exhaustion, was not supported. A closer look at Table 4 shows that the main effects of RC level on emotional exhaustion (*p* < 0.01) and work engagement (*p* < 0.01) were significant. Moreover, the analysis yielded a non-significant main effect of emotion regulation as a covariate for both emotional exhaustion (*p* = ns) and work engagement (*p* = ns).

**Table 4:** Results of MANCOVA: RC intensity level on work engagement and emotional exhaustion.

**Source**	**Dependent variable**	**df**	***F***	***p***	**Eta**
Intercept	WE	1	12.60	0.001	0.19
	EEX	1	28.42	0.000	0.34
Emotion regulation	WE	1	0.38	0.54	0.007
	EEX	1	0.37	0.55	0.007
RC level	WE	1	112.23	0.001^**^	0.65
	EEX	1	103.71	0.001^**^	0.65
Conflict management style	WE	1	12.41	0.001^**^	0.18
	EEX	1	25.60	0.001^**^	0.318
RC level × conflict management style	WE	1	11.72	0.001^**^	0.18
	EEX	1	0.85	0.362	0.02
Error	WE	55			
	EEX	55			
Total	WE	60			
	EEX	60			

*N = 60*,

** *p = 0.01*,

^*^p = 0.05, two-tailed; RC, Relationship Conflict; WE, Work Engagement; EEX, Emotional Exhaustion.

Follow-up univariate analyses for RC level and conflict management style on emotional exhaustion and work engagement were performed separately. The results were identical to that of the MANCOVA; as such, the interactive term for work engagement was significant (*p* < 0.01), and that for emotional exhaustion was nonsignificant (*p* < 0.05, ns).

As an additional analysis, we conducted a moderation analysis to test whether emotion regulation moderates the relation between RC and emotional exhaustion and work engagement. The results show that distraction, but not cognitive reappraisal, moderates the association between high RC and emotional exhaustion (B = −0.46, *t* = −0.2.69, *p* < 0.01, 95% confidence interval [CI]: −0.81; −0.12). Furthermore, distraction and cognitive reappraisal did not moderate the link between RC and work engagement.

### Discussion

Consistent with previous research (Dijkstra et al., 2009; Benitez et al., 2018), design 1 indicates a positive relationship between RC and emotional exhaustion and a negative relationship with work engagement (Buric and Macuka, 2018). The results also show differing effects of RC level on emotional exhaustion and work engagement. At a high level of RC (vs. a low level), more emotional exhaustion and less work engagement were reported. An explanation might be that heightened RC reveals more animosity (Jehn, 1995), and elevated conflict is more likely to evoke devastative reactions (Ayoko et al., 2008). Applying the attentional resource model (Ellis and Ashbrook, 1988) when RC is prevalent in workgroups, group members allocate psychological and emotional resources to address personal issues, reducing attentional resources for engaging at work tasks. This reduces the resources available for work engagement (Chen et al., 2011).

The results demonstrate that a cooperative conflict management style moderates the relationship between RC and work engagement but not emotional exhaustion. This finding can be attributed to the fact that using a cooperative style, which is positive and unlikely to be emotionality charged, may facilitate engaging at work. As Medina and Benitez (2011) show, a cooperative style emphasizes shared interests, which may lessen the intensity of high RC. Thus, consistent with past research, a cooperative style is an appropriate and effective tool for handling the adverse effects of RC (Gross and Guerrero, 2000) on work engagement.

Contrary to our expectations, a cooperative conflict management style was not effective for moderating the detrimental effects of high RC on emotional exhaustion. One possible reason is that RC creates an imbalance between the emotional demands of the situation and the resources available to meet such demands. A cooperative style may, thus, be less able to call on emotional resources to reduce RC. This finding falls in line with studies in a different context. For example, Benitez et al. (2018) show that using a passive (avoidant) conflict management style could mitigate the effects of RC on emotional exhaustion. Such findings suggest that the effectiveness and appropriateness of a cooperative style (vs. competitive) in moderating the effects of RC could depend upon the intensity of RC and type of workgroup outcomes.

Concerning our additional analysis, our results show that distraction moderates the link between RC and emotional exhaustion, which is consistent with past research (Griffith et al., 2014; Esbati and Korunka, 2020). As previously discussed, one reason is that distraction requires relatively simple processes and blocks emotional involvement before it accumulates. This reduces susceptibility to negative emotions and provides the opportunity for recovering and gaining required resources. The finding suggests that distraction could be an appropriate and effective means of moderating the negative effects of RC on emotional exhaustion.

## DESIGN 2: Task Conflict

### Results

As in design 1, design 2 varied a high vs. low conflict and cooperative vs. competitive strategy in a 2 × 2 between-subjects design. Means, SD, and correlations were calculated to provide a general understanding of the relationship between the variables of interest (Tables 5, 6). The means and SD according to the conditions for TC, work engagement, and emotional exhaustion are presented in Table 7.

**Table 5:** Descriptive statistics, and correlations coefficients for the study variables.

**Variables**	***M***	**SD**	***n***	**1**	**2**	**3**
1. Emotional exhaustion	3.67	1.01	60	-		
2. Work engagement	4.10	0.61	60	−0.52^**^	**-**	
3. Emotion regulation	4.58	0.83	60	−0.38^**^	0.33^*^	**-**

** *p = 0.01*,

* *p = 0.05, two tailed*.

**Table 6:** Descriptive statistics for task conflict and conflict management style (*N* = 60).

**Variables**		**Task conflict**		**Conflict management style**	
		**Low**	**High**	**Cooperative**	**Competitive**
Emotional exhaustion	M	3.67	4.59	3.92	4.28
	SD	0.86	0.92	1.04	0.97
Work engagement	M	4.04	3.31	4.00	0.41
	SD	0.42	0.53	3.35	0.61

**Table 7:** Descriptive statistics according to the conditions for work engagement and emotional exhaustion (*N* = 60).

**Levels of Task conflict**	**Conflict management style**	**Emotional exhaustion**		**Work engagement**	
		***M***	**SD**	***M***	**SD**
Low	Cooperative	3.53	0.94	4.24	0.34
	Competitive	3.70	0.81	3.83	0.41
High	Cooperative	4.31	1.03	3.74	0.31
	Competitive	4.87	0.73	2.87	0.29

As Table 6 illustrates, high-TC participants perceived significantly higher emotional exhaustion than did low-TC participants [*F* = (1, 58) = 17.77, *p* = 0.001]. Compared with their high-TC counterparts, low-TC participants also reported higher work engagement [*F* = (1, 58) = 34.44, *p* = 0.001]. The findings, thus, demonstrate that the high-TC participants perceived more emotional exhaustion and less work engagement than did the low-TC participants. Linear regression indicates that the TC level is associated with emotional exhaustion (β = 0.48, *t* = 4.22, *p* = 0.001) and work engagement (β = −0.61, *t* = −5.87, *p* = 0.001). These findings support H2 and H4. Table 7 indicates that in the conditions of low levels of TC and using a cooperative style, the mean of work engagement (*M* = 4.24, SD = 0.34) is greater than high levels of TC (*M* = 3.74, SD = 0.31).

**Figure 3:**
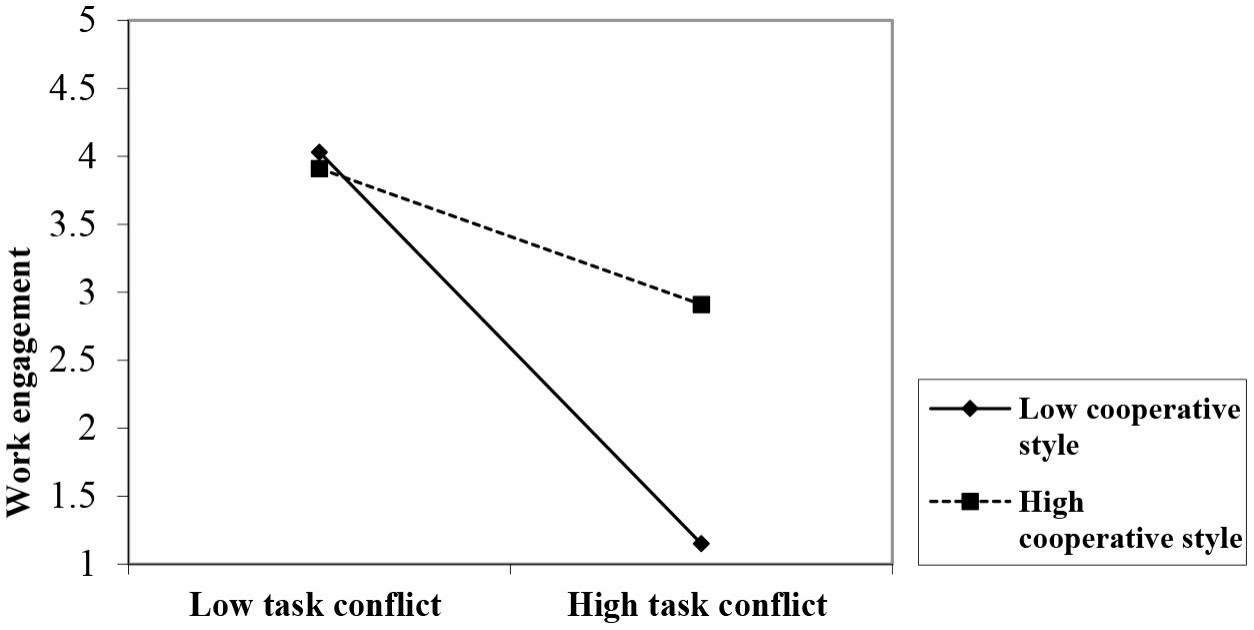
The moderating effect of conflict management style on the relationship between task conflict (TC) and work engagement.

As in design 1, we conducted MANCOVA and ANCOVA to assess the influence of TC level (low vs. high) and cooperative vs. competitive style on emotional exhaustion and work engagement. The results illustrate a significant interaction of TC level and conflict management style on work engagement [Table 8, *F* = (1, 55) = 8.04, *p* = 0.006]. Our results confirm the moderating role of a cooperative style on work engagement, supporting H8. The graphical presentation of the interaction was derived using a standard regression coefficient of the regression lines for workgroup high and low (±1 SD of the mean) on the moderator variable of a cooperative style (Figure 3). At high levels of TC, the workgroup members' cognitive and emotional loads and tension increase, thereby drawing resources away from the processing of information associated with critical debates of differing perspectives. This may block an open and constructive discussion and create reluctance and withdrawal, and a cooperative style requires tackling conflict actively and openly. However, the results demonstrate that the interaction term of TC level and conflict management style on emotional exhaustion was non-significant [*F* = (1, 55) = 0.94, *p* > 0.05]. Thus, H6, proposing that a cooperative style moderates the relationship between TC level and emotional exhaustion, was not confirmed. The related findings show that the main effects of TC level on emotional exhaustion [*F* = (1, 55) = 11.59, *p* < 0.01] and work engagement [*F* = (1, 55) = 54.29, *p* < 0.01] were significant. Emotion regulation, as a covariate, showed a significant main effect on emotional exhaustion [*F* = (1, 55) = 4.84, *p* < 0.05] and work engagement [*F* = (1, 55) = 4.47, *p* < 0.05].

**Table 8:** Results of MANCOVA: TC intensity level on work engagement and emotional exhaustion.

**Source**	**Dependent variables**	**df**	***F***	***p***	**Eta**
Intercept	WE	1	149.18	0.001	0.73
	EEX	1	69.49	0.001	0.56
Emotion regulation	WE	1	4.47	0.03^*^	0.08
	EEX	1	4.84	0.03^*^	0.08
TC level	WE	1	54.29	0.001^**^	0.50
	EEX	1	11.59	0.001^**^	0.17
Conflict management style	WE	1	57.79	0.001^**^	0.51
	EEX	1	2.87	0.01^**^	0.05
TC level × conflict management style	WE	1	8.04	0.006^**^	0.13
	EEX	1	0.94	0.34	0.02
Error	WE	55			
	EEX	55			
Total	WE	60			
	EEX	60			

*N = 60*,

** *p = 0.01*,

* *p = 0.05, two tailed; TC, Task Conflict; WE, Work Engagement; EEX, Emotional Exhaustion*.

Follow-up univariate analyses for TC level and conflict management style on emotional exhaustion and work engagement were performed separately. The results were identical to that of the MANCOVA; thus, the interactive term for work engagement was significant (*F* = 1, 56 = 7, 25, *p* < 0.01, η = 0.12, observed power = 0.75), and that for emotional exhaustion was non-significant (*p* > 0.05, ns).

Concerning our additional analysis, the results show that distraction and cognitive reappraisal did not moderate the association between TC level and emotional exhaustion and work engagement.

### Discussion

Consistent with prior research (De Dreu and Weingart, 2003; De Dreu, 2008), design 2 indicates that working under high-TC conditions increases emotional exhaustion. Congruent with the few related studies examining the association between TC and work engagement (e.g., Selmer et al., 2013; Jungst and Blumberg, 2016), our study also shows that TC reduces work engagement.

Our findings illustrate that high TC (vs. low) impairs group members' emotional and physical potential and inhibits them from engaging at work. The findings suggest that increased task disagreements disrupt information processing and divert a group member's attention from fellow workgroup members' views. Rigidly holding onto an initial view prevents group members from engaging at work. Consequently, heightened TC leads to cognitive rigidity, defensiveness, and withdrawal (Carnevale and Probst, 1998). This, in turn, escalates conflict, leading to reduction of the group members' energy and reduces group effort (O'Neill and McLarnon, 2018).

In our investigation, conflict management style moderated the relationship between TC level and work engagement but not emotional exhaustion. One plausible reason for this is that handling task disagreements cooperatively enhances self-efficacy and satisfaction as well as harmony in the workgroup (De Dreu, 2008). This, in turn, encourages group members to discuss task issues openly. They can then become more confident that their workgroup opponents are pursuing mutually beneficial solutions. This would conserve cognitive and emotional resources to buffer the negative effects of TC on work engagement and unfreeze the information sharing process (e.g., Hobfoll, 1991).

The results illustrate that a competitive conflict management style does not buffer the effects of TC on emotional exhaustion and work engagement. Perhaps negative emotions escalate when group members view TC as a win–lose struggle. Hence, deadlocks prevail, and group members block each other's efforts (e.g., Tjosvold et al., 2003).

## General Discussion

The main purpose of this research was to examine the distinct effects of RC and TC intensity (low vs. high) on emotional exhaustion and work engagement. To do so, we decoupled RC and TC and examined them in two separate experimental, scenario-based designs. The other purpose of our research was to explore the moderating effects of cooperative and competitive conflict management styles in the link between RC (and TC) and emotional exhaustion and work engagement. To rule out the effects of emotion regulation (via distraction and cognitive reappraisal), we assessed it as a covariate.

The results support the premise that higher levels of both RC and TC are positively related to emotional exhaustion and negatively related to work engagement. This finding is consistent with the study of Ayoko et al. (2008), who demonstrate that conflict intensity is significantly associated with destructive reactions to conflict. Past research has also documented the adverse influences of RC on emotional exhaustion (Benitez et al., 2018; Esbati and Korunka, 2020) and work engagement (Chen et al., 2011; Buric and Macuka, 2018). The main reason for this may be that RC context generates emotional exhaustion, burnout (Schaufeli and Salanova, 2014), and work disengagement (Jungst and Blumberg, 2016) due to increased tension and social and psychological discomfort (Seo et al., 2004). These feelings deplete energy and interfere with the group members' cognitive functioning needed to engage at work.

The results of this study reveal that a cooperative conflict management style is an appropriate and effective means of mitigating the detrimental effects of heightened RC and TC on work engagement but not emotional exhaustion. This could imply that, in the context of heightened conflict, the benefit of using a cooperative style may be limited to attenuate the adverse effects of conflict on work engagement. Perhaps, there is no match between the emotional exhaustion and a cooperative style. The key component in emotional exhaustion is emotions, and conflict management has mainly cognitive components. This aligns with the triple-match principle (De Jonge and Dormann, 2006) proposing that stressors, moderators, and the outcomes should be qualitatively similar to produce a buffering/moderator effect. Supporting this implication, Bear et al. (2014) reveal that an avoidant conflict management style mitigated the relationship between negative emotions engendered by RC and emotional exhaustion. Similarly, Benitez et al. (2018) find that avoiding and integrating conflict management styles buffered the link between RC and collective emotional exhaustion.

Consistent with past research (Griffith et al., 2014; Esbati and Korunka, 2020), our additional analysis reveals that distraction moderates the link between RC and emotional exhaustion. This suggests that there may be a match between RC, the distraction strategy, and emotional exhaustion: emotions play a critical role in each of them. This match could produce a moderator effect.

The results could clarify the mixed findings for TC (De Dreu and Weingart, 2003; De Wit et al., 2012). Intensity (low vs. high) may account for the negative effects of TC in some studies (De Dreu and Weingart, 2003). This claim is consistent with some studies that have stated “intensity” of conflict leads to adverse effects on outcomes such as innovation and creativity (De Dreu, 2006). Participants at low levels of TC were less likely to make personal judgments about other group members' opinions, and it was less likely for these workgroups to react emotionally to cognitive debate. Our results further imply that high RC generates detrimental effects on workgroup outcomes.

Our research makes a number of contributions to the intragroup conflict literature. The vast majority of studies on the effects of intragroup conflict are correlational with all the limitations of inferring causation from correlational research (e.g., Calder et al., 1981). We conducted scenario-based experiments that allowed us to examine the true causal relationships between variables while controlling confounding variables. We recruited participants who were employed and who had work experiences in a real workgroup setting. In contrast to prior research on conflict that focused mainly on distal outcomes, in particular, performance, our research provides evidence about how and when TC and RC may be associated with emotional exhaustion and work engagement. By experimentally disentangling TC and RC occurrence and manipulating the intensity of conflict expression (low vs. high), we employed a new approach to investigating conflict and directly examined the assumption that more TC and RC lead to more emotional exhaustion and less work engagement compared with low RC and TC. This may add new insights in the literature concerning the effects of intragroup conflict.

### Limitations and Future Research

The limitations in the present study should be acknowledged. The first limitation revolves around the group sample. We used a convenience sample, which imposes limits on generalizability. Additionally, our sample was highly educated, which may imply comparatively low reactivity to conflicts. Our findings should be replicated using samples more representative of the employed population. Furthermore, the participants were mostly from multinational companies, and they lived and worked in Austria. Factors such as culture (i.e., collectivist vs. individualist) and workgroup norms could color the way intragroup conflict is viewed and managed (e.g., Chen et al., 2016). For instance, workgroup members might already deem what conflict management styles are (in)appropriate. However, it was beyond of the scope of our study. Future work in this area needs to address this issue. However, this limitation was controlled through assigning participants randomly to one of eight conditions. The second limitation revolves around the conflict management styles. Given our study design, we assessed two conflict management styles although group members might adopt more than two conflict management styles depending on the situation (Van de Vliert, 1997). According to the contingency approach, there is no best way to respond to conflict. Thus, the effectiveness of conflict management style in response to conflict may vary with different contexts of conflict intensity. Future study may consider more than two styles of conflict management. The third limitation revolves around emotion regulation, which was assessed as a covariate. Given the role of distraction in handling conflict (Griffith et al., 2014), future studies may manipulate distraction to explore its moderating role in the context of intragroup conflict.

Although our research is not exempt from limitations, it provides useful inputs for clarifying the findings on intragroup conflict. Furthermore, our study offers some insight into effective tools for managing conflict and enhancing well-being in a workgroup.

### Theoretical and Practical Implications

Our results have several theoretical and practical implications. From a theoretical standpoint, our results demonstrate that decoupling RC and TC and assessing their intensity are vital for clarifying their effects on workgroup outcomes. Our results support the argument that TC (De Dreu, 2006) and RC could produce curvilinear effects.

Our findings have important implications for managers. Managers need to assess the intensity of conflict in their workgroups to ensure that their efforts to motivate their workgroup members are not nullified by dysfunctional group dynamics. Managers who encounter workgroups with high levels of intragroup conflict should mitigate its effects on employees' well-being by training them in conflict management and even emotion regulation strategies. The results also suggest that different types of conflict need to be managed differently. Therefore, any training program for managing intragroup conflict needs to be comprehensive and offer the required skills for conflict management and emotion regulation strategies.

## Conclusion

Despite the obvious limitations, the study enriches the conflict literature by examining the interplay between intragroup conflict and conflict management. The key contributions of this study to the literature include decoupling the theoretical and methodological conflict types (task and relationship), examining conflict features (intensity), and handling conflict and its effects on emotion exhaustion and work engagement. Decoupling the types of conflict aided the isolation of specific effects of conflict on our study outcomes. The manipulation of conflict intensity and the way it is managed also offered better understanding of the connection between the variables of interest.

## Data Availability Statement

The raw data supporting the conclusions of this article will be made available by the authors, without undue reservation.

## Ethics Statement

The studies involving human participants were reviewed and approved by The Institutional Review Board of the department of Work, Economy, and Social Psychology, University of Vienna. Written informed consent for participation was not required for this study in accordance with the national legislation and the institutional requirements.

## Author Contributions

ZE and CK contributed to the conception and design of the study and read and edited the manuscript during the preparation of the manuscript. ZE organized the collection, preparation of data, performed the statistical analyses, and wrote the first draft of the manuscript. Both authors contributed to the article and approved the submitted version.

## Conflict of Interest

The authors declare that the research was conducted in the absence of any commercial or financial relationships that could be construed as a potential conflict of interest.

## Appendix

**Appendix A:** Selected statements used in the scenarios.

**Relationship conflict**	
Low	These differences sometimes easily lead to exchanges that are slightly heated and emotional.
High	The exchanges frequently turn into quarrels and became personal, nasty, and disrespectful.
**Task conflict**	
Low	Each member's ideas are slightly challenged in heated discussions, but are almost always respectful.
High	Each member's ideas are always challenged in heated discussions, but are almost always respectful.

## References

[B1] Andrews I. R. Tjosvold D. (1983). Conflict management under different levels of conflict intensity. J. Occup. Behav. 4, 223–228.

[B2] Ayoko O. B. Callan V. J. Härtel C. E. J. (2008). The influence of team emotional intelligence climate on conflict and team members' reactions to conflict. Small Group Res. 39, 121–149. 10.1177/1046496407304921

[B3] Bakker A. B. Demerouti E. (2007). The job demands-resources model: state of the art. J. Manag. Psych. 22, 309–328. 10.1108/02683940710733115

[B4] Bear J. B. Weingart L. R. Todorova. (2014). Gender and the emotional experience of relationship conflict: the differential effectiveness of avoidant conflict management. Nego. Conflict Manage. Res. 7, 213–231. 10.1111/ncmr.12039

[B5] Behfar K. J. Peterson R. S. Mannix E. A. Trochim W. M. (2008). The critical role of conflict resolution in teams: a close look at the links between conflict type, conflict management strategies, and team outcomes. J. Appl. Psychol. 93, 170–188. 10.1037/0021-9010.93.1.170 18211143

[B6] Benitez M. Medina F. Munduate L. (2018). Buffering relationship conflict consequences in teams working in real organizations. Internal. J. Conflict Manage. 29, 279–297. 10.1108/IJCMA-11-2017-0131

[B7] Brown S. P. Leigh T. W. (1996). A new look at psychological climate and its relationship to job involvement, effort, and performance. J. Appl. Psychol. 81, 358–368. 10.1037/0021-9010.81.4.358 8751453

[B8] Bruk-Lee V. Nixon A. E. Spector P. E. (2013). An expanded typology of conflict at work: task, relationship and non-task organizational conflict as social stressors. Work Stress. 27, 339–350. 10.1080/02678373.2013.841303

[B9] Buric I. Macuka I. (2018). Self-Efficacy, emotions and work engagement among teachers: a two wave cross-lagged analysis. J. Happiness Study. 19, 1917–1933. 10.1007/s10902-017-9903-9

[B10] Calder B. J. Phillips L. W. Tybout A. M. (1981). Designing research for applications. J. Consumer Res. 8, 197–207. 10.1086/208856

[B11] Carnevale P. J. Probst T. M. (1998). Social values and social conflict in creative problem solving and categorization. J. Pers. Soc. Psychol. 74, 1300–1309. 10.1037/0022-3514.74.5.1300

[B12] Chen A. S. Y. Hou Y. H. Wu I. H. (2016). Handling conflict at work–the impact of active and agreeable conflict styles. Internal. J. Conflict Manage. 27, 50–61. 10.1108/IJCMA-10-2014-0076

[B13] Chen Z. Zhang X. Vogel D. (2011). Exploring the underlying processes between conflict and knowledge sharing: a work engagement perspective. J. Appl. Soc. Psychol. 41, 1005–1033. 10.1111/j.1559-1816.2011.00745.x

[B14] Costa P. Passos A. M. Bakker A. B. (2014). Teamwork engagement: a model of emergence. J. Occup. Organiz. Psychol. 87, 414–436. 10.1111/joop.12057

[B15] Costa P. L. Passos A. M. Bakker A.B. (2015). Direct and contextual influence of team conflict on team resources, teamwork engagement, and team performance. Nego. Conflict Manage. Res. 8, 211–227. 10.1111/ncmr.12061

[B16] Curşeu P. Boroş S. Oerlemans L. (2012). Task and relationship conflict in short-term and long-term groups: the critical role of emotion regulation. Intern. J. Conflict Manage. 23, 97–107. 10.1108/10444061211199331

[B17] De Dreu C. K. W. (2006). When too little or too much hurts: evidence for a curvilinear relationship between task conflict and innovation in teams. J. Manage. 32, 83–107. 10.1177/0149206305277795

[B18] De Dreu C. K. W. (2008). The virtue and vice of workplace conflict: food for (pessimistic) thought. J. Organiz. Behav. 29, 5–18. 10.1002/job.474

[B19] De Dreu C. K. W. Beersma B. (2005). Conflict in organizations: beyond effectiveness. Euro. J. Work Organiz. Psychol. 14, 105–117. 10.1080/13594320444000227

[B20] De Dreu C. K. W. Weingart L. (2003). Task versus relationship conflict, team performance, and team member satisfaction: a meta-analysis. J. Appl. Psychol. 88, 741–749. 10.1037/0021-9010.88.4.741 12940412

[B21] De Jong A. Song M. Song L. Z. (2013). How lead founder personality affects new venture performance: the mediating role of team conflict. J. Manage. 39, 1825–1854. 10.1177/0149206311407509

[B22] De Jonge J. Dormann C. (2006). Stressors, resources, and strain at work: a longitudinal test of the triple-match principle. J. Appl. Psychol. 91, 1359–1374. 10.1037/0021-9010.91.5.1359 17100490

[B23] De Wit F. R. C. Greer L. L. Jehn K. A. (2012). The paradox of intragroup conflict: a meta-analysis. J. Appl. Psychol. 97, 360–390. 10.1037/a0024844 21842974

[B24] DeChurch L. A. Mesmer-Magnus J. R. Doty D. (2013). Moving beyond relationship and task conflict: toward a process-state perspective. J. Appl. Psychol. 98, 559–578. 10.1037/a0032896 23731027

[B25] Demerouti E. Bakker A. Nachreimer F. Schaufeli W. (2001). The job demands resources model of burnout. J. Appl. Psychol. 86, 499–512. 10.1037/0021-9010.86.3.499 11419809

[B26] Dickerson S. S. (2008). Emotional and physiological responses to social-evaluative threat. Soc. Pers. Psychol. Compass. 2, 1362–1378. 10.1111/j.1751-9004.2008.00095.x25264711

[B27] Dickerson S. S. Gruenewald L. T. Kemeny E. M. (2004). When the social self is threatened: shame, physiology, and health. J. Personal. 72, 1191–1216. 10.1111/j.1467-6494.2004.00295.x 15509281

[B28] Dijkstra M. T. M. Beersma B. Evers A. (2011). Reducing conflict related employee strain: the benefits of an internal locus of control and a problem-solving conflict management strategy. Work Stress 2, 167–184. 10.1080/02678373.2011.593344

[B29] Dijkstra M. T. M. De Dreu C. K. W. Evers A. Van Dierendonck D. (2009). Passive responses to interpersonal conflict at work amplify employee strain. Eur. J. Work Organ. Psychol. 18, 405–423. 10.1080/13594320802510880

[B30] Dijkstra M. T. M. Van Dierendonck D. Evers A. (2005). Responding to conflict at work and individual well-being: the mediating role of flight behaviour and feelings of helplessness. Euro. J. Work Organ. Psychol. 14, 119–135. 10.1080/13594320444000254

[B31] Ellis H. C. Ashbrook P. W. (1988). Resource allocation model of the effects of depressed mood states on memory, in Affect, Cognition and Social Behaviour, ed. Fiedler K Forgas J (Toronto, ON: Hogrefe), 25–43.

[B32] Esbati Z. Korunka C. (2020). What moderates the relation between intragroup conflict, emotional exhaustion, and work management? Scand. J. Work Organ. Psychol. 5, 1–15. 10.16993/sjwop.91

[B33] Friedman R. A. Tidd S. T. Currall S. C. Tsai J. C. (2000). What goes around comes around: The impact of personal conflict style on work group conflict and stress. Intern. J. Conflict Manage. 10, 17–35. 10.1108/eb022834

[B34] Galbraith J. R. (1974). Organization design: an information processing view. Interfaces 4, 28–36. 10.1287/inte.4.3.28

[B35] Griffith J. A. Connelly S. Thiel C. E. (2014). Emotion regulation and intragroup conflict: when more distracted minds prevail. Intern. J. Conflict Manag. 25, 148–170. 10.1108/IJCMA-04-2012-0036

[B36] Gross J. J. (1998). Antecedent- and response-focused emotion regulation: divergent consequences for experience, expression, and physiology. J. Pers. Soc. Psychol. 74, 224–237. 10.1037/0022-3514.74.1.224 9457784

[B37] Gross J. J. (2001). Emotion regulation in adulthood: timing is everything. Curr. Direct. Psych. Sci. 10, 214–219. 10.1111/1467-8721.00152

[B38] Gross J. J. (2002). Emotion regulation: affective, cognitive and social consequences. Psychophysiology. 39, 281–291. 10.1017/S0048577201393198 12212647

[B39] Gross J. J. John O. P. (2003). Individual differences in two emotion regulation processes: implications for affect, relationships, and well-being. J. Personal. Soc. Psychol. 85, 348–362. 10.1037/0022-3514.85.2.348 12916575

[B40] Gross M. Guerrero L. (2000). Managing conflict appropriately and effectively: an application of the competence model to Rahim's organizational conflict management styles. Intern. J. Conflict Manag. 11, 200–226. 10.1108/eb022840

[B41] Hayes A. F. (2012). E. coli. Process: A Versatile Computational Tool for Observed Variable Mediation, Moderation, and Conditional Process Modelling. Available online at: http://afhayes.com/public/process2012.pdf (accessed March 18, 2019).

[B42] Hobfoll S. E. (1991). Traumatic stress: a theory based on rapid loss of resources. Anxiety Res. 4, 187–197. 10.1080/08917779108248773

[B43] Huynh J. Y. Xanthopoulou D. Winefield A. H. (2014). The Job demands-resources model in emergency service volunteers: examining the mediating roles of exhaustion, work engagement and organizational connectedness. Work Stress 28, 305–322. 10.1080/02678373.2014.936922

[B44] Janssen O. Devliert E. Veenstra C. (1999). How task and person conflict shape the role of positive interdependence in management teams. J. Manage. 25, 117–142. 10.1177/014920639902500201

[B45] Jehn K. (1995). A multimethod examination of the benefits and detriments of intragroup conflict. Admin. Sci. Q. 40, 256–282. 10.2307/2393638

[B46] Jehn K. Mannix E. (2001). The dynamic nature of conflict: a longitudinal study of intragroup conflict and group performance. Acad. Manage. J. 44, 238–251. 10.5465/3069453

[B47] Jehn K. A. (1997). A qualitative analysis of conflict types and dimensions in organizational groups. Admin. Sci. Q. 42, 530–557. 10.2307/2393737

[B48] Jehn K. A. Bendersky C. (2003). Intragroup conflict in organizations: A contingency perspective on the conflict-outcome relationship. Res. Org. Behav. 25, 89–244. 10.1016/S0191-3085(03)25005-X

[B49] Jehn K. A. Greer L. Levine S. Szulanski G. (2008). The effects of conflict types, dimensions, and emergent states on group outcomes. Group Dec. Nego. 17, 465–495. 10.1007/s10726-008-9107-0

[B50] Jiang J. Y. Zhang X. Tjosvold D. (2013). Emotion regulation as a boundary condition of the relationship between team conflict and performance: a multi-level examination. J. Organiz. Behav. 34, 714–734. 10.1002/job.1834

[B51] John O. P. Gross J. J. (2004). Healthy and unhealthy emotion regulation: personality processes, individual differences, and life span development. J. Pers. 72, 1301–1334. 10.1111/j.1467-6494.2004.00298.x 15509284

[B52] Jungst M. Blumberg B. (2016). Work relationships: counteracting the negative effects of conflict. Intern. J. Confl. Manag. 27, 225–248. 10.1108/IJCMA-10-2014-0079

[B53] Korsgaard M. A. Jeong S. S. Mahony D. M. Pitariu A. H. (2008). A multilevel view of intragroup conflict. J. Manag. 34, 1222–1252. 10.1177/0149206308325124

[B54] Kozlowski S. W. J. Bell B. S. (2013). Work groups and teams in organizations, in Handbook of Psychology: Industrial and Organizational Psychology, eds Weiner I. B. Schmitt N. W. Highhouse S. (Hoboken, NJ: Wiley), 412–469.

[B55] Kuriakose V. Seejesh S. Wilson P. R. Mr A. (2019). The differential association of workplace conflicts on employee well-being: the moderating role of perceived social support at work. Intern. J. Confl. Manag. 30, 1044–1068. 10.1108/IJCMA-05-2018-0063

[B56] Lazarus R. S. Alfred E. (1964). Short-circuiting of threat by experimentally altering cognitive appraisal. J. Abnorm. Soc. Psychol. 69, 195–205. 10.1037/h0044635 14213291

[B57] Maslach C. Jackson S. E. (1981). The measurement of experienced burnout. J. Occup. Behav. 2, 99–113. 10.1002/job.4030020205

[B58] Matta F. K. Erol-Korkmaz H. T. Johnson R. E. Biçaksiz P. (2014). Significant work events and counterproductive work behavior: the role of fairness, emotions, and emotion regulation. J. Organ. Behav. 35, 920–944. 10.1002/job.1934

[B59] Medina F. J. and Benitez, M. (2011). Effective behaviors to decrease the intensity of escalated conflict in organizations. In Benitez, M., Medina, F. and Munduate, L. (2018). Buffering relationship conflict consequences in teams working in real organizations. Int. J. Conflict Manage. 29, 279–297.

[B60] Meier L. L. Gross S. Spector P. E. Semmer N. K. (2013). Relationship and task conflict at work: interactive short-term effects on angry mood and somatic complaints. J. Occup. Health Psych. 18, 144–156. 10.1037/a0032090 23506551

[B61] O'Neill T. A. McLarnon M. J. W. (2018). Optimizing conflict dynamics for high performance teamwork. Human Resou. Manag. Rev. 28, 378–394. 10.1016/j.hrmr.2017.06.002

[B62] Pruitt D. G. Rubin J. (1986). Social Conflict: Escalation, Stalemate and Settlement. New York, NY: Random House.

[B63] Quick J. C. Quick J. D. Nelson D. L. Hurrel J. J (1997). Preventive Stress Management in Organizations. Washington, DC: American Psychological Association. 10.1037/10238-000

[B64] Rahim M. A. (2002). Toward a theory of managing organizational conflict. Intern. J. Confl. Manag. 13, 206–235. 10.1108/eb022874

[B65] Schaufeli W. Bakker A. (2004). Job demands, job resources, and their relationship with burnout and engagement: a multi-sample study. J. Organ. Behav. 25, 293–315. 10.1002/job.248

[B66] Schaufeli W. B. Leiter M. P. Maslach C. Jackson S. E. (1996). The Maslach Burnout inventory – general survey, in Burnout Inventory manual, 3rd ed., ed Maslach C. Jackson S. E. Leiter Maslach M. P. (Palo Alto, CA: Consulting Psychologists Press), 191–218.

[B67] Schaufeli W. B. Salanova M. (2014) Burnout, boredom engagement in the workplace, in People at Work: An Introduction to Contemporary Work Psychology, Peeters M. C. W. de Jonge J. Toon W. Taris T. W. (Chichester: Wiley Blackwell), 293–320.

[B68] Schaufeli W. B. Salanova M. González-Rom V. Bakker A. B. (2002). The measurement of engagement and burnout: a two sample confirmatory actor analytic approach. J. Happiness Stud. 3, 71–92. 10.1023/A:1015630930326

[B69] Selmer J. Jonasson C. Lauring J. (2013). Group conflict and faculty engagement: is there a moderating effect of group trust? J. Higher Educ. Policy Manag. 35, 95–109. 10.1080/1360080X.2013.748477

[B70] Seo M. G. Barrett L. F. Bartunek J. M. (2004). The role of affective experience in work motivation. Acad. Manag. Rev. 29, 423–439. 10.5465/amr.2004.13670972 16871321 PMC1519413

[B71] Shaw J. D. Zhu J. Duffy M. K. Scott K. L. (2011). A contingency model of conflict and team effectiveness. J. Appl. Psychol. 96, 391–400. 10.1037/a0021340 20939655

[B72] Sheppes G. Meiran N. (2007). Better late than never? On the dynamics of on-line regulation of sadness using distraction and cognitive reappraisal. Pers. Soc. Psychol. Bull. 33, 1518–1532. 10.1177/0146167207305537 17933748

[B73] Sheppes G. Scheibe S. Suri G. Gross J. J. (2011). Emotion-regulation choice. Psychol. Sci. 22, 1391–1396. 10.1177/0956797611418350 21960251

[B74] Sheppes G. Scheibe S. Suri G. Radu P. Blechert J. Gross J. J. (2014). Emotion regulation choice: a conceptual framework and supporting evidence. J. Experi. Psychol. 143, 163–181. 10.1037/a0030831 23163767

[B75] Solansky S. T. Singh B. Huang S. (2014). Individual perceptions of task conflict and relationship conflict. Nego. Confl. Manag. Res. 7, 83–89. 10.1111/ncmr.12027

[B76] Spaapen D. L. Brummer L. Stopa L. Waters F. Bucks R. S. (2014). The emotion regulation questionnaire: validation of the ERQ-9 in two community samples. Psychol. Assess. 26, 46–54. 10.1037/a0034474 24059476

[B77] Spector P. E. Bruk-Lee V. (2008). Conflict, health, and well-being, in The Psychology of Conflict and Conflict Management in Organizations, eds De Dreu C. K. W. Gelfand M. J. (San Francisco, CA: Jossey-Bass), 267–288.

[B78] Staw B. M. Sandelands L. E. Dutton J. E. (1981). Threat-rigidity effects on organizational behaviour. Admin. Sci. Q. 26, 501–524. 10.2307/2392337

[B79] Stewart L. G. Barrick M. R. (2017). Team structure and performance: assessing the mediating role of intrateam process and the moderating role of task type. Acad. Manag. J. 43, 135–148. 10.5465/1556372

[B80] Tekleab A. G. Quigley N. R. Tesluk P. E. (2009). A longitudinal study of team conflict, conflict management, cohesion, and team effectiveness. Group Organ. Manag. 34, 170–205. 10.1177/1059601108331218

[B81] Tjosvold D. Hui C. Ding D. Hu J. (2003). Conflict values and team relationships: Conflict's contribution to team effectiveness and citizenship in China. J. Organ. Behav. 24, 69–88. 10.1002/job.180

[B82] Van de Vliert E. (1997). Complex Interpersonal Conflict Behaviour: Theoretical Frontiers. Hove: Psychology Press.

