# Supplementary material for: Does Intragroup Conflict Intensity Matter? The Moderating Effects of Conflict Management on Emotional Exhaustion and Work Engagement
Source: Front Psychol. 2021 May 31;12:614001. doi: 10.3389/fpsyg.2021.614001 (PMC8202687; doi:10.3389/fpsyg.2021.614001)
Supplement: Supplementary file 1 [file Data_Sheet_1.pdf]

Supplementary Material

Scenario One

Situation: Please read the description below carefully and imagine yourself in the situation described.

Suppose you and four other people are all members of the same company workgroup. You are all working full-time and are all at the same rank in the company. All of you have been meeting twice a week to coordinate your shared tasks for different projects. Everyone has noticed that you all frequently disagree with each other about how to manage the projects. You and the other view the projects very differently, and all of you strongly advocate your own opinions. Most of the time in workgroup meetings is spent discussing recent problems facing the projects and how to solve them. Each of you propose very different solutions. Each member's ideas are usually challenged in heated discussions but are almost always respectful. Phrases such as "We cannot afford that." and "We tried that, and it didn't work." are common. Each of you often thinks that the others' proposals are risky or naive, and that implementing them would seriously hamper success of your projects. Over time your workgroup has tried different ways to resolve its many disagreements. Most often the disagreements are resolved by combining your diverse ideas into a group's consensus. Each of you believes that to resolve a disagreement you should address each of members concerns in the project as best as you can. You all try to integrate the views of everyone involved.

Scenario Two

Situation: Please read the description below carefully and imagine yourself in the situation described.

Suppose you and four other people are all members of the same company workgroup. You are all working full-time and are all at the same rank in the company. All of you have been meeting twice a week to coordinate your shared tasks for different projects. Everyone has noticed that you all frequently disagree with each other about how to manage the projects. You and the other view the projects very differently, and all of you strongly advocate your own opinions. Most of the time in workgroup meetings is spent discussing recent problems facing the projects and how to solve them. Each of you propose very different solutions. Each member's ideas are usually challenged in heated discussions but are almost always respectful. Phrases such as "We cannot afford that." and "We tried that, and it didn't work." are common. Each of you often thinks that the others' proposals are risky or naive, and that implementing them would seriously hamper success of your projects. Over time members of your workgroup most often resolve disagreements by forcing their views on others. Shouting matches are common. Each of you believes that your own view outweighs the perspective of the others, and argues his/her own position more vigorously until the others give in.

Scenario Three

Situation: Please read the description below carefully and imagine yourself in the situation described.

Suppose you and four other people are all members of the same company workgroup. You are all working full-time and are all at the same rank in the company. All of you have been meeting twice a week to coordinate your shared tasks in different projects. Everyone has noticed that you all occasionally disagree about how to manage the projects. You and the others view the projects occasionally differently, and all of you sometimes advocate your own opinions. Most of the time in workgroup meetings is spent discussing recent problems facing the projects and how to solve them. Each of you propose slightly different solutions. Each member's ideas are sometimes challenged in heated discussion discussions but are almost always respectful. Phrases such as " We cannot afford that." and "We tried that, and it didn't work." are

occasionally heard. Now and then a group member strongly insists on his/her work opinion on the issues. Each of you occasionally thinks that the others' proposals are risky or naive, and that implementing them would seriously hamper the success of your projects. Over time your workgroup has tried different ways to resolve its many disagreements. Most often the disagreements are resolved by combining your diverse ideas into a group's consensus. Each of you believes that to resolve a disagreement you should address each of members concerns in the project as best you can. You all try to integrate the views of everyone involved.

Scenario Four

Situation: Please read the description below carefully and imagine yourself in the situation described.

Suppose you and four other people are all members of the same company workgroup. You are all working full-time and are all at the same rank in the company. All of you have been meeting twice a week to coordinate your shared tasks in different projects. Everyone has noticed that you all occasionally disagree about how to manage the projects. You and the others view the projects occasionally different, and all of you sometimes advocate your own opinions. Most of the time in workgroup meetings is spent discussing recent problems facing the projects and how to solve them. Each of you propose slightly different solutions. Each member's ideas are sometimes challenged in heated discussion discussions but are almost always respectful. Phrases such as " We cannot afford that." and "We tried that and it didn't work." are occasionally heard. Now and then a group member strongly insists on his/her work opinion on the issues. Each of you occasionally thinks that the others' proposals are risky or naive, and that implementing them would seriously hamper the success of your projects.Over time members of your workgroup most often resolve disagreements by forcing their views on others. Shouting matches are common. Each of you believes that your own view outweighs the perspective of the others, and argues his/her own position more vigorously until the others give in.

Scenario Five

Situation: Please read the description below carefully and imagine yourself in the situation described.

Suppose you and four other people are all members of the same company workgroup. You are all working full-time and are all at the same rank in the company. Everyone has noticed that you dislike one another. You and the others have very different preferences, work styles and personalities. For example, some of you always prefer open windows in the office, while others prefer keeping the windows closed; two of your work group members frequently arrive at least 15 minutes late to the meetings, while the rest of you arrive on time and wait. These differences often easily lead to exchanges that are often heated and emotional. The exchanges frequently turn into quarrels and become personal, nasty and disrespectful. Phrases such as "You are a jerk." "You are an idiot" are frequently heard. Over time your workgroup has tried different ways to resolve its many disagreements. Most often the disagreements are resolved by combining your diverse ideas into a group's consensus. Each of you believes that to resolve a disagreement you should address each of members concerns as best you can. You all try to integrate the views of everyone involved.

Scenario Six

Situation: Please read the description below carefully and imagine yourself in the situation described.

Suppose you and four other people are all members of the same company workgroup. You are all working full-time and are all at the same rank in the company. Everyone has noticed that you dislike one another. You and the others have very different preferences, work styles and personalities. For example, some of you always prefer open windows in the office, while others prefer keeping the windows closed; two of your work group members frequently arrive at least

15 minutes late to the meetings, while the rest of you arrive on time and wait. These differences often easily lead to exchanges that are often heated and emotional. The exchanges frequently turn into quarrels and become personal, nasty and disrespectful. Phrases such as “You are a jerk.” “You are an idiot” are frequently heard. Over time members of your workgroup most often resolve disagreements by forcing their views on others. Shouting matches are common. Each of you believes that your own view outweighs the perspective of the others, and argues his/her own position more vigorously until the others give in.

Scenario Seven

Situation: Please read the description below carefully and imagine yourself in the situation described.

Suppose you and four other people are all members of the same company workgroup. You are all working full-time and are all at the same rank in the company. Everyone has noticed that some of you dislike one another. You and the others have slightly different preferences, work styles and personalities. For example, some of you sometimes prefer open windows in the office, while others prefer keeping the windows closed; two of your work group members occasionally arrive at least 15 minutes late to the meetings, while the rest of you arrive on time and wait. These differences sometimes easily lead to exchanges that are slightly heated and emotional. The exchanges sometimes turn into quarrels and became personal, nasty and disrespectful. Phrases such as “You are a jerk.” “You are an idiot” are sometimes heard. .Over time your workgroup has tried different ways to resolve its many disagreements. Most often the disagreements are resolved by combining your diverse ideas into a group’s consensus. Each of you believes that to resolve a disagreement you should address each of members concerns as best you can. You all try to integrate the views of everyone involved.

Scenario Eight

Situation: Please read the description below carefully and imagine yourself in the situation described.

Suppose you and four other people are all members of the same company workgroup. You are all working full-time and are all at the same rank in the company. Everyone has noticed that some of you dislike one another. You and the others have slightly different preferences, work styles and personalities. For example, some of you sometimes prefer open windows in the office, while others prefer keeping the windows closed; two of your work group members occasionally arrive at least 15 minutes late to the meetings, while the rest of you arrive on time and wait. These differences sometimes easily lead to exchanges that are slightly heated and emotional. The exchanges sometimes turn into quarrels and became personal, nasty and disrespectful. Phrases such as “You are a jerk.” “You are an idiot” are sometimes heard. .Over time members of your workgroup most often resolve disagreements by forcing their views on others. Shouting matches are common. Each of you believes that your own view outweighs the perspective of the others, and argues his/her own position more vigorously until the others give in.
